# Evolving perspectives on the sources of the frequency-following response

**DOI:** 10.1038/s41467-019-13003-w

**Published:** 2019-11-06

**Authors:** Emily B. J. Coffey, Trent Nicol, Travis White-Schwoch, Bharath Chandrasekaran, Jennifer Krizman, Erika Skoe, Robert J. Zatorre, Nina Kraus

**Affiliations:** 1 Department of Psychology, Concordia University, 1455 Boulevard de Maisonneuve Ouest, Montréal, QC, H3G 1M8 Canada; 2 International Laboratory for Brain, Music, and Sound Research (BRAMS), Montréal, QC Canada; 3 Centre for Research on Brain, Language and Music (CRBLM), McGill University, 3640 de la Montagne, Montréal, QC H3G 2A8 Canada; 4 Auditory Neuroscience Laboratory, Department of Communication Sciences, Northwestern University, 2240 Campus Dr., Evanston, IL 60208 USA; 5 Communication Sciences and Disorders, School of Health and Rehabilitation Sciences, University of Pittsburgh, Forbes Tower, 3600 Atwood St, Pittsburgh, PA 15260 USA; 6 Department of Speech, Language, and Hearing Sciences, The Connecticut Institute for the Brain and Cognitive Sciences, University of Connecticut, 2 Alethia Drive, Unit 1085, Storrs, CT 06269 USA; 7 Montreal Neurological Institute, McGill University, 3801 rue Université, Montréal, QC H3A 2B4 Canada; 8 Department of Neurobiology, Northwestern University, 2205 Tech Dr., Evanston, IL 60208 USA; 9 Department of Otolaryngology, Northwestern University, 420 E Superior St., Chicago, IL 6011 USA

**Keywords:** Auditory system, Learning and memory, Neural ageing, Sensory processing

## Abstract

The auditory frequency-following response (FFR) is a non-invasive index of the fidelity of sound encoding in the brain, and is used to study the integrity, plasticity, and behavioral relevance of the neural encoding of sound. In this Perspective, we review recent evidence suggesting that, in humans, the FFR arises from multiple cortical and subcortical sources, not just subcortically as previously believed, and we illustrate how the FFR to complex sounds can enhance the wider field of auditory neuroscience. Far from being of use only to study basic auditory processes, the FFR is an uncommonly multifaceted response yielding a wealth of information, with much yet to be tapped.

## Abstract (web summary)

The auditory frequency-following response (FFR) indexes the quality of neural sound encoding in the brain. In this Perspective, the authors discuss the potential of the FFR to provide a better understanding of sound encoding in the auditory system and its relationship to behavior.

## Introduction

The auditory system must faithfully encode and process rapid variations in acoustic signals and precisely extract important features, such as frequency, amplitude modulation, and sound onsets and offsets. This task is accomplished by a complex, interconnected, and parallel system. Auditory information enters the brainstem from the cochlea via the auditory nerve and ascends via both lemniscal and nonlemniscal auditory pathways^1^. Neurons in the lemniscal (or “primary/classical”) pathway are thought to be the main bearers of temporally varying information, with synapses in the brainstem (cochlear nucleus and superior olivary complex), midbrain (central nucleus of the inferior colliculus), thalamus (ventral division of the medial geniculate body), and the primary auditory cortex. The fidelity of sound encoding in these ascending pathways affects all cognitive processes that use the information—and in turn, these ascending pathways are affected by cognitive processes via the vast efferent system. Consequently, sound encoding is relevant to the study of many higher-level functions central to human communication, including speech and music.

Frequency-following responses (FFRs) are recordings of phase-locked neural activity that is synchronized to periodic and transient aspects of sound. Traditionally, FFRs have been measured in humans as electrophysiological potentials to sound, recorded from the scalp. For guidance on collecting FFRs, see Skoe and Kraus for a tutorial in EEG-FFR collection^2^, Krizman and Kraus for a tutorial on EEG-FFR analysis^3^, and Coffey et al. for technical details on the MEG-FFR^4^ (see Box 1 for key points).

Human FFRs were first measured in the 1970s^5^. Identified as subcortical in origin, they were viewed as a potential supplement to behavioral audiometry. Over the years, the field has moved away from treating the subcortical auditory system as a bottom-up, hardwired conduit for sound, and is increasingly recognizing the contribution of top-down influences within the context of distributed neural networks. Studies using the FFR have played an instrumental role in this evolution of thinking.

**Fig. 1:**
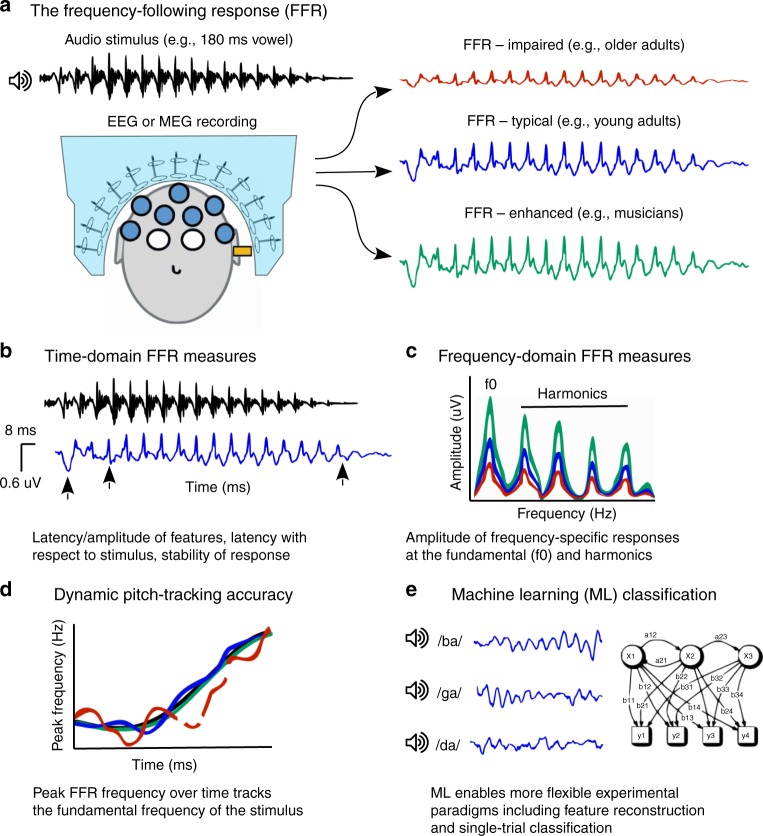
The FFR is a means of non-invasively measuring the brain’s ability to encode sound, as well as the general integrity of the auditory system. **a** The FFR is measured using EEG or MEG while periodic or quasi-periodic sounds such as vowels, consonant-vowel syllables, or tones are presented (see also Box 1). The morphology of the averaged evoked response differs between individuals as a function of pathology and expertize. FFRs can be visualized in **b** the time domain, **c** the frequency domain, and **d** as the accuracy of changes in frequency content over time in response to spectrally dynamic stimuli. **e** Classification accuracy derived from machine learning techniques provides an additional metric

The FFR is a noninvasive means of reliably measuring the fidelity and precision with which the brain encodes sound. Measures derived from the FFR (e.g. timing, amplitude, consistency, and pitch tracking, see Fig. 1) reveal an individual’s mapping between a stimulus and the brain’s activity, which may be impaired in disease or enhanced through expertize. The FFR has proven essential to answering basic questions about how our auditory system manages complex acoustic information, how it integrates with other senses, and how both tasks are shaped by experience^6–8^. FFR measures are related to the ability to differentiate sounds, hear targets in noise, and to experience with music, tonal languages, or multilingualism^8–13^. The FFR can reveal the plastic nature of the human auditory system, including its potential to change over short-time scales, and its sensitivity to enriched and impoverished experiences with sound^13–22^.

The FFR is useful to address questions concerning impaired auditory processing in populations with impaired cochlear function^23–26^, and in neurodevelopmental speech and language disorders^27–32^ or autism^33,34^. It can also be used to study maturational^35,36^ and aging-related changes^37,38^, sex differences in auditory functions^39^, and improvement caused by interventions^15,40–42^. More broadly, the FFR can provide an index of neurological health, for instance, in populations with acquired neurological disorders (e.g. concussion)^43^. For a comprehensive review of FFR and its role in indexing the effects of experience on the auditory brain, see refs. ^44,45^.

A fundamental question is what source(s) underlie the FFR in humans. This is important for basic scientific knowledge for its own sake and also because a greater understanding of the FFR’s sources can inform its translation and deployment in medicine. Methods have emerged that allow for some spatial separation of FFR sources in humans (i.e., brainstem, thalamus, cortex^4,46,47^). These studies have reopened questions about the degree to which activity in different subcortical and cortical centres contributes to the well-studied scalp-recorded FFR and whether sources identified using other methods generalize to the traditional, scalp-recorded response. To be clear: while many questions remain to be answered, we do not think the FFR is solely generated in the auditory cortex, nor do we exclude the possibility of cortical contributions under certain circumstances.

Here we aim to update our evolving understanding of the FFR in a way that is accessible to an interdisciplinary audience; and, we wish to outline a roadmap that promotes a more integrative understanding of the FFR and its potential to study human auditory function.

## Historical roots and changing views

To our knowledge, the term “frequency-following response” was dubbed in the late 1960s by Worden and Marsh^48^, where it was described in an animal model. Initially investigated with low-frequency pure tones (<500 Hz), FFRs were an appealing alternative/adjunct to other objective measures of auditory function available at the time (e.g., auditory brainstem responses, electrocochleograms) because the latter have poor frequency specificity and are less effective at evoking responses to stimulus frequencies below 500 Hz.

By the 1990s, however, evidence began to emerge that the FFR reflected more than mere stimulus audibility. Gary Galbraith, a pioneer in the use of richer FFR stimuli such as two-tone complexes, missing fundamental stimuli, and speech, reported that the FFR was affected by attention^49^ and by how a particular speech stimulus was perceived by the listener^50^. Galbraith’s insight that “the FFR is a unique tool for understanding the most important of all auditory capacities: the coding and processing of human language” has proven prescient as the 21st century has seen a dramatic increase in investigations into speech-evoked FFR and how response properties relate to human communication. With these discoveries has come a renewed interest in the investigation of the FFR above and beyond its ability to signal sound detection. Instead, as we detail below, the FFR is now seen as a powerful tool to understand the neurophysiological bases of complex auditory behaviors in humans, including speech and music.

Evoked responses, which are also derived from EEG recordings but typically using a low-pass frequency filter (<40 Hz, often referred to as “cortical auditory evoked potentials” or “late-latency responses” and their variants, such as the mismatch negativity or P300), generally reflect a response to stimulus onset and later processing stages. Distinguishing the FFR is the precision with which it retains the morphological features of the waveform of the stimulus, therefore revealing how the auditory system responds to its acoustic elements. An uncommon wealth of analysis strategies accompanies interpretation of this multifaceted response (see Fig. 1 and Box 2). The past 10 years have seen refinements of FFR analyses that capitalize on the richness of the response^3^.

## Evidence for multiple sources in human scalp-recorded FFR

The biological sources of the FFR have been a topic of debate since the early days of the technique^51–53^. Efforts to clarify the sources of far-field responses have yielded greater understanding of how and where auditory information is integrated across auditory and non-auditory regions and timescales, and the degree to which auditory centres are subject to neuroplasticity^54,55^.

Our view of the FFR’s origins relies on three axioms about the auditory system.

1. The central auditory system is a network of intertwined structures that extend across medulla, pons, midbrain, thalamus, and temporal lobes of cortex. This network is intrinsically connected to other sensory systems and motor, cognitive, and reward systems. To be sure, cells and circuits within each of the nuclei have specialized functions and properties; but, none of these cells or circuits operates in a vacuum. The interactivity of the system means that even something as simple as a primary auditory cortex neuron’s tuning curve has to be considered within the broader context of an integrative and plastic system (reviewed in Kraus and White-Schwoch^44^). Thus, any consideration of one or more sources of the FFR also has to consider how those sources interact with each other and with non-auditory brain circuits. It is also important to bear in mind that the same auditory structure can yield different neural activity depending on the sound’s context^29,56–58^.
2. Phase-locking, the phenomenon by which neurons discharge at a particular phase within the stimulus cycle, is a common feature throughout the auditory system. Through this action the recurring, periodic elements of the stimulus (e.g., the period of the fundamental frequency, the period of the amplitude modulation frequency) are encoded in the synchronous activity of a neuronal population. As you ascend the lemniscal pathway the rate of phase-locking decreases. (For more on auditory system phase-locking see Box 3 and Fig. 2).

**Fig. 2:**
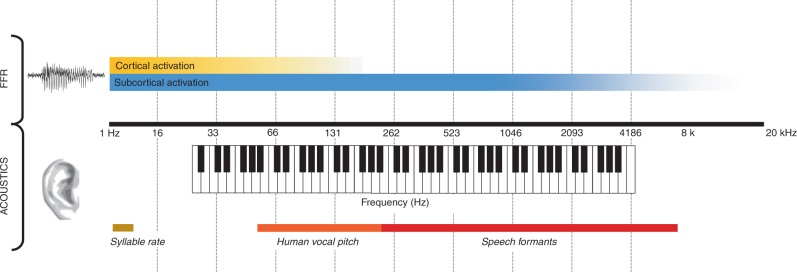
Schematic of frequency ranges of speech and music and the relative activation of subcortical and cortical phase-locking to the frequency-following response. Phase-locking limitations of neurons and neuronal assemblies in the human auditory system are not yet known, but can be partly inferred from animal models. Despite phase-locking limitations, the frequency-following response is predictive of the functionality of the entire auditory system
3. The auditory system is plastic. Neurons throughout the auditory axis exhibit rapid plasticity based on stimulus context (e.g., Carbajal and Malmierca^59^) and the interactive nature of the auditory system makes each centre subject to non-auditory input, whether by changes in overall brain physiology or metabolism, changes in environmental input, and/or changes in top-down cognitive input to refine sensory representation. Thus, while an FFR might measure the current functional state of stimulus representation in the auditory brain, that functional state reflects the legacy of this plasticity.

### What supports the conventional wisdom that the FFR has a subcortical origin?

Our current understanding of sources of human scalp-recorded FFR is the culmination of non-invasive studies in humans and invasive studies in animal models, each of which has advantages and limitations. The inferior colliculus has often been considered as the dominant source of the FFR derived from EEG scalp-recordings (EEG-FFR) (reviewed in Chandrasekaran and Kraus^60^), based on the auditory system’s reduced capacity for high-frequency phase-locking at higher centres. Additional evidence comes from direct recordings in animal models, in which the neural sources of the FFR have been studied by selectively taking different auditory structures offline by cooling, lesioning, or pharmacological manipulation. For example, the scalp-recorded FFR was abolished or strongly reduced by cryogenic blockade of the IC in cats^51^, and in human patients with focal IC lesions^52^, confirming that the IC is an important FFR signal generator. While these experiments ruled out more peripheral sources, they cannot rule out thalamic or cortical sources—since the IC is an obligatory station of the afferent pathway, blocking IC activity fails to disambiguate IC vs. thalamocortical contributions. Approaching this question from the other direction, studies in cats and rabbits showed that FFRs close to 100 Hz remained largely unaffected by decreased auditory cortex function, but were influenced by lesions to the inferior colliculus^61^. Also noteworthy is that speech-evoked FFRs and evoked responses to amplitude-modulated tones recorded directly from subcortical structures in animals strongly resemble those recorded from the brain’s surface and those recorded to the same stimuli in humans^62,63^.

The FFR’s short stimulus-to-response latency of ~5–9 ms is often quoted as evidence of a subcortical origin (e.g. ref. ^64^), as the IC has a latency of 5–7 ms. However, latency-based arguments are difficult to defend as FFR latencies vary considerably according to stimulus characteristics such as sound pressure level, frequency, and amplitude envelope, and stimulus-to-response latencies much longer than 7 ms have been reported between the stimulus and EEG-FFR in some studies (e.g. 14.6 ms^65^). Furthermore, intracranial recordings from Heschl’s gyrus show that the first responses to sound in the cortex can occur as early as ~9 ms post stimulus onset^66^.

### Rethinking FFR sources: The multiple generator hypothesis

There have long been hints of the idea that the FFR comprises multiple generators. We advance the hypothesis that the EEG-FFR is an aggregate response reflecting multiple auditory stations, including the auditory nerve, cochlear nucleus, inferior colliculus, thalamus, and cortex, and that the specific mixture of sources may vary depending on the recording techniques, stimulus, and participant demographic. This hypothesis motivates several predictions.

**Fig. 3:**
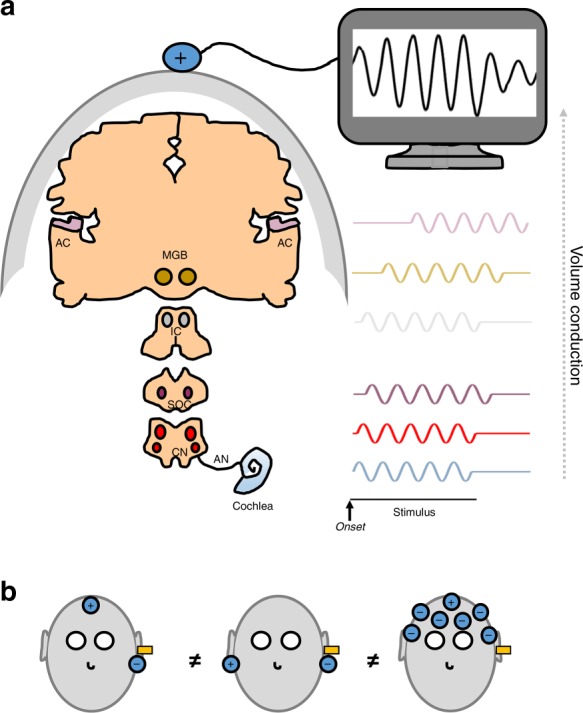
**a** Scalp-recorded frequency-following responses (FFRs) may reflect, in part, the summation of phase-locked activity from different sources, each with a characteristic lag relative to the onset of the stimulus. The putative sources of the FFRs include the cochlea, auditory nerve (AN), cochlear nucleus (CN), superior olive (SOC), inferior colliculus (IC), medial geniculate body (MGB), and auditory cortex (AC). **b** Electrode montage influences the relative contribution of sources in the scalp-recorded signal: for example, the montages shown on the left and central panels which include an electrode at the mastoid likely include a greater contribution from peripheral sources than does the montage illustrated on the right, which references a single vertex channel to the average of other scalp electrodes
- Prediction 5: Different recording techniques will differ in their sensitivity to different sources. Source-localized EEG-FFR and MEG-FFR do not show identical patterns of source strength^4,46^. Results from MEG should not be directly applied to EEG due to their differing sensitivities to radial vs. tangential currents, and to superficials vs. deep sources (discussed with reference to FFR in ref. ^4^); although they both are sensitive to the electrochemical current flows within and between brain cells, they provide partly overlapping and partly complementary information^73–75^. Still, even using only EEG-FFR, electrode placement and referencing appears to affect signal content. Coffey et al. compared two common electrode montages and found only a moderate correlation in their sensitivity to behavioral measures^76^; these montages, often used interchangeably, may thus differ in the combination of sources to which they are sensitive (Fig. 3b). Likewise, reaction times on an auditory task were noted to track with amplitude of the EEG-FFR in an electrode montage that favors more central subcortical sources, but not in responses from a simultaneously recorded montage that was more peripherally biased^77^.

- Prediction 1: Decomposition of a multichannel EEG signal should indicate multiple, independent components. In 1978, Stillman et al. recorded FFRs to tones with various fundamental frequencies using only two EEG channels, and concluded that the human FFR is a composite of several waveforms whose relative influence differs as a function of frequency^53^. Kuwada et al. recorded human EEG and electrophysiology in rabbits and concluded that surface recordings are composite responses from multiple brain generators^62^. Two-channel recordings and principal component analysis on multichannel EEG data have demonstrated separable FFR components that relate to stimulus properties, such as the presence or absence of energy at the fundamental frequency^64,67,68^.
- Prediction 2: Multimodal source modeling should indicate multiple generators of the scalp-recorded signal. Coffey et al. reported that FFRs to speech (with *f*0 ~100 Hz) could be non-invasively recorded using MEG, which allows spatial source localization. MEG-FFR contributions included not only subcortical sources—the cochlear nucleus, inferior colliculus, and medial geniculate body (thalamus)—but also the auditory cortices (with a right-hemisphere predominance)^4^. Using a combination of EEG and functional magnetic resonance imaging (fMRI), a subsequent study confirmed that hemodynamic activity in the right auditory cortex was related to individual differences in the EEG-based FFR *f*0 strength, consistent with the hypothesis that phase-locked activity in auditory cortex has a hemodynamic signature^69^. Bidelman found corroborating evidence of multiple sources to the FFR, including a cortical one, using distributed source modeling techniques on multichannel EEG recordings and a speech stimulus (with *f*0 in the same range as in Coffey et al.). This EEG approach revealed subcortical sources contributing more than the auditory cortex^46^ (note that thalamic sources did not appear to be included in the analysis).
- Prediction 3: Individual differences in FFR components should correlate with behavior if they are functionally relevant. Zhang and Gong used principal component analysis on multichannel EEG data, and found multiple, separable components with different scalp topographies, only one of which correlated with pitch perception; they concluded that phase-locked activity at different sources differentially relates to behavior^68^. Coffey et al. observed significant correlations between the magnitude of the right auditory cortical MEG-FFR response and pitch perception thresholds, as well as with musical training, suggesting that phase-locked activity in this region provides behaviorally–relevant information^4^. Separately, while the MEG-FFR strength at subcortical and cortical sources was predictive of speech-in-noise (SIN) perception, the strongest correlations were observed with the right auditory cortex^70^. In a cross-modal attention task, Hartmann and Weisz confirmed the strong contribution of cortical regions to the MEG-FFR and found that only the right auditory cortex was significantly affected by attention^71^.
- Prediction 4: Different stimulus frequencies will bias certain generators. Tichko and Skoe conducted an extensive investigation that measured EEG-FFR amplitude to complex tones as a function of fundamental frequency^72^. EEG-FFRs to stimuli with frequencies between 16.35 and 880 Hz showed generally decreasing amplitude with increasing frequency, but with local maxima at ~44, 87, 208, and 415 Hz. The local maxima suggest an EEG-FFR with multiple underlying generators whose activity interacts constructively or destructively at the scalp depending on the stimulus frequency (Fig. 3a). The EEG-FFR interference pattern that produced these local maxima was modeled by the authors as the summation of multiple phase-locked signals, all phase-locked to the stimulus frequencies but with different latencies (i.e., neural conduction times). The authors suggested that recording protocol, electrode montage, recording quality (i.e. signal-to-noise ratio), and subject demographics influence the EEG-FFR interference patterns because each one of these manipulations alters the strength of phase-locking or the degree to which this phase-locking can be detected at the scalp.

A thread through this work is that recording modalities, stimuli, and stimulus presentation paradigms all may influence the mix of sources underlying the recorded signal. One must therefore exercise caution in extrapolating conclusions from one modality or paradigm to the results of another.

In summary, the extent of contributions of sources to the scalp-recorded EEG-FFR under different experimental conditions and in different populations is an unsettled topic. Yet the discovery that different recording techniques implicate different underlying generators increases the richness of what FFR can tell us. We find ourselves sympathetic to the view that the EEG-FFR signal can represent a mixture of sources including the auditory nerve, CN, IC, MGB, and cortex, and that the contribution of each source could differ depending on where and how the response is recorded. Regardless of the “real-time” sources of an FFR, and the possibility that one source may dominate the response, we want to reemphasize that each of those potential sources operates in concert with each other (and non-auditory systems) to shape its function.

### Approaches to test hypotheses about FFR origins

To make further progress on these concepts, it will be useful to employ methods whereby FFR data are collected simultaneously with other data that unambiguously reflect cortical and network activity^70,78–80^. Functional connectivity measures that allow for quantification of the strength and direction of information transfer may also prove useful when applied to spatially resolved signals such as EEG/MEG in source space^81^. Combinations of different methods could be especially valuable, such as EEG-based FFR together with fMRI or functional near-infrared spectroscopy (fNIRS)^69,82^. fMRI or fNIRS provides a means of quantifying functional networks throughout the brain which could be used to relate to FFR variables.

Recent animal neurophysiology studies have demonstrated that an FFR similar to that of humans can be recorded in awake monkeys^83^, confirming previous demonstrated analogs between humans and anesthetized non-human animals^37,38,84,85^. Awake animal preparations could be particularly enlightening because of the possibility of recording simultaneously from multiple sites in behaving animals. Neurophysiological studies in animals and humans^86^ could provide a ground truth comparison for FFR strength estimates, establishing cellular-level correlates of observable EEG signals and their changes with plasticity. Another approach would be to combine FFR measurements with brain stimulation of the auditory cortex.

There is also still more to learn about the “old-fashioned” scalp-recorded FFR. Much work to date has focused on the lower-frequency components of the response relating to the fundamental frequency of the stimulus, even though there are approaches that bias responses to high-frequency cues such as speech formants. A wealth of analysis techniques accompanies the interpretation of the FFR; see Krizman and Kraus^3^. A deeper understanding of these FFR components can enrich our understanding of complex auditory behaviors. And, when applied in tandem with animal research and other techniques, these techniques can further our understanding of generators underlying these relatively simple paradigms.

Finally, new methods to collect FFR offer many interesting possibilities for future research. For example, an exciting future direction is to record FFR to continuous, natural speech or other signals, instead of the traditional repeated singles stimulus paradigm^87–90^. Combined with free-field recordings^91^, portable FFR systems^92^, and/or wearable technologies^93^, these methods open opportunities to examine FFR in real-world settings. On the analytical front, machine learning algorithms have recently been developed allowing single-trial FFR classification^94,95^ which could have many applications, including for instance as neurofeedback in training paradigms.

### Network dynamics and the “functional view” of the FFR

Contemporary approaches in systems and cognitive neuroscience emphasize the concept that the nervous system functions as an integrated set of complex networks, comprising various interconnected nodes and hubs at which distinct operations take place^96^, and from whose interactions complex cognition emerges. This perspective strongly informs our view that the auditory nervous system exhibits extensive bidirectional cortical–subcortical and ipsilateral–contralateral connectivity (in addition to bidirectional connectivity with other sensory and cognitive systems). In turn, auditory cortex may itself be considered a hub^97^ for the ventral and dorsal corticocortical loops that are known to underwrite auditory cognition including auditory object recognition, localization, speech, and music^98–101^. Thus, we may consider the entire auditory system as consisting of a number of conjoined complex networks, each of which is of course far from fully characterized at this point.

Taking this idea of a highly interconnected nervous system as a framework, we suggest that the FFR serves as an index of the functional properties of the subcortical and early cortical parts of the auditory system. By virtue of the interconnectedness of networks, the FFR is a snapshot of auditory processing. It also seems that the FFR would be influenced by, and hence be relevant to, the corticocortical loops as well. Although direct evidence for such network-level influences remains sparse, the modulation of FFR parameters associated with training-induced plasticity or with cortical dysfunction, as mentioned above, may be one instantiation of this phenomenon^15,102^. Similarly, the proposals that the FFR may be influenced by attention^71,103–105^ (but see ref. Varghese et al.^106^), arousal state^107^, or task demands^76,86,108^, may constitute another example. Conceptually similar is the idea that stimulus-specific adaptation (and mismatch negativity) were originally considered cortical^109^, but we now know that they reflect an integrated auditory change detection response^56,57,110,111^.

It is our view that the FFR should be thought of as an aggregate measure of the response of the auditory system, reflecting its cumulative prior history. Specific auditory brain centres may contribute differently to a measured response, but those centres function jointly, and in the context of broader neural networks. This gives us the “functional view” of the FFR—we see it as a measure of how well the entire brain is coding sound features much more than as a reflection of activity within any single nucleus, because the nuclei are embedded in complex functional networks. Distinct computations may happen at local nodes, but the functional metrics can be considered as an emergent property of the interactions between nodes. Considering the FFR in this way leads to the development of systems-level hypotheses that should encourage understanding of the relationships between the FFR and other neural features. For example, combining FFR measures with functional MRI may prove useful in delineating the interactions between auditory representations and higher-order cognitive functions (e.g., attention, memory, and even visual and motor operations) and how these interactions change with experience. Similarly, functional and structural connectivity metrics offer opportunities to explore individual differences in network properties and how they affect auditory encoding. All of these approaches can also inform questions relating to development and maturation, as well as to aging and disorders.

Box 1 FFR collection and analysis The FFR may be measured in its most simple form using one-channel EEG, with a reasonably high sampling rate (>2000 Hz) and open filters, as subjects are presented with a high number of repetitions (500+) of an auditory stimulus such as a complex tone or speech syllable. FFRs have commonly been measured to stimuli with fundamental frequencies above 80 Hz. Although frequency tracking occurs in the brain at much lower frequencies (e.g. ~2–4 Hz^112^) it remains to be seen whether these lower frequencies are tracked by identical neural mechanisms. The most commonly used electrode montages are single or averaged earlobes (or mastoids) to vertex (Cz), or hairline (~FCz) to 7th cranial vertebra (see Fig. 3b). More recently, multichannel EEG, and MEG, and complementary measures of whole-brain hemodynamic response (BOLD fMRI)^69^ have been used to provide spatial information. In most studies to date, the stimulus has a fundamental frequency between 80 and 500 Hz. Stimulus duration is usually between 40 and 250 ms, representing a trade-off between ecological validity and expedience, as high numbers of repetitions are needed. However, even sentence-length stimulation has been used^88^.

Insert tube earphones are used to avoid electromagnetic stimulus artifacts, and stimulation is generally suprathreshold (70–80 dB SPL). Stimuli may be presented monaurally (e.g. to study lateralization^113^) or binaurally (to maximize the amplitude of the FFR). They are often presented in alternating polarity (i.e. the digital signal is multiplied by −1 for half of the presentations), which allows for analysis of both envelope and spectral responses.

Experimental approaches should be matched to the research questions and constraints. For research questions that concern the function and contribution of specific structures or localize individual differences, methods that provide spatio-temporal separation are necessary (e.g. MEG). For research questions concerning identification of biomarkers, relation to behavioral performance, or tracking longitudinal changes, simpler equipment with a lower cost is desirable. The single-EEG channel FFR has repeatedly proven its worth as a sensitive measure of the fidelity with which the auditory system as a whole preserves useful sound information^6,45^. Intermediate approaches that make use of information from more than one channel^64,114^ or stimulus are also possible. For example, results from several studies measuring FFRs to a range of frequencies suggested frequency-dependent cortical contributions, thus generating hypotheses for future studies with more definitive localization methodology^47,72,103^.

Box 2 Terminology A source of confusion in the field stems from the many terms and acronyms used to refer to the FFR (e.g. ABR, sABR, cABR, AMFR, SS-EP, SSR). The auditory brainstem response has been invoked to help ground FFR in a known clinical technique. Thus, speech-evoked ABR (or “sABR”) will turn up in some circles. Kraus and colleagues promoted “cABR” for some time. The “c” stands for complex, referring to the evoking stimulus, to contrast it with the clicks and pure tones of conventional audiometric ABR. It was also employed to avoid limiting the discussion of the response to phase-locking to the stimulus periodicity (i.e., the following of the stimulus frequency); cABR was meant to be inclusive of the transient onsets and offsets present in the recording. However, we no longer use the term—in part due to its potentially misleading emphasis on “brainstem,” in addition to the implication that it has identical sources and generating mechanisms as the ABR. Another term used is “envelopefollowing response”^115^. For speech stimuli, however, the term is suboptimal because it incorrectly implies that temporal fine structure (TFS, the counterpart to the acoustic envelope) cannot be observed in the response. Although the acoustic envelope, which imparts important perceptual attributes of sound (e.g., voice pitch), is the most widely studied facet of the FFR, other facets that have received attention include the TFS and responses to distortion products^3,116,117^. We propose that terms such as envelope and temporal fine structure be subordinated as modifiers: FFR_ENV_ and FFR_TFS_. The rarely used “amplitude modulation following response”, likewise, is too limiting, suggesting a narrowly defined stimulus type^62^. SS-EP, steady-state evoked potential^7^, and SSR, steady-state response^114^ have also emerged recently, adding to the alphabet soup of terminology. The lack of standardized terminology frustrates literature searches, creates false impressions, and at times leads to unnecessary balkanization among researchers. We advocate for simply calling it “FFR,” which avoids presupposing a single source, emphasizing one aspect of the response over another, and limiting the stimulus set. FFR, while imperfect, is a transparent name because it emphasizes that it is a neural response that reflects the acoustical properties of the inducing stimulus, including its periodic and non-periodic components. From a practical standpoint, the term FFR lends itself to easy modification based on collection technique (EEG-FFR, MEG-FFR), analysis technique (FFR_ENV_, FFR_TFS_) or response subcomponent of interest (FFR-F0, FFR-harmonics). And, it has been the modal term for the past 50 years.

Box 3 Phase-locking in the auditory system As a general principle, as you ascend the auditory system, neural response latency increases, phase-locking (i.e. neurons discharging at a particular phase within the stimulus cycle) becomes weaker at higher stimulus frequencies, and the frequency range over which phase-locking occurs becomes narrower. It is known from single-neuron recordings that although the upper limit of stimulus synchronization declines progressively in the ascending pathway from 5000 Hz in the auditory nerve, it is still present up to 800 Hz in the medial geniculate body^118–120^, with some reports suggesting thalamic phase-locking as high as 1000 Hz in cats^121^. While the upper limit of phaselocking of individual cortical neurons is often quoted as ~100 Hz, this limit may be an underestimate as it has not clearly been established in humans. Intracranial recording directly from the auditory cortex is possible in neurosurgical epilepsy patients, and using this method, clear phase-locked near-field responses have been reported in response to click trains up to 200 Hz^122,123^ and to speech stimuli with fundamental frequencies within the range of 120 Hz^86^. The volley principle^124^, that multiple neurons each contribute to a subdivision of a period, in theory, could enable even higher frequencies to be coded by neural populations. Indeed, multiple-neuron activity recordings from the monkey primary auditory cortex show phase-locking capability at 250 Hz frequencies (but not at 500 Hz)^125^, and in the guinea pig some auditory cortex units phase locked up to 500 Hz (with some units tuned specifically to the fundamental frequency range of the species’ 270 Hz alarm calls, suggesting behavioral importance^126^). The overlap in the phaselocking ranges observed across the auditory neuraxis together with the contribution of the response to transient stimulus components create the physiological conditions for the FFR for any given stimulus frequency to have multiple potential underlying generators. The presence of multiple sources creates complex far-field waveforms that, because of the phase relationships of the sources, can lead to false conclusions about the number and latency of the sources^52,72,127^.

Box 4 Questions for future research

- Why do FFRs, which cover only a relatively narrow band of frequencies compared to the entire hearing range (Fig. 2a), nonetheless predict such a wide range of auditory behaviors?
- How does the FFR relate to other measures that are sensitive to quasi-periodic or aperiodic fluctuations in the signal that allow for reconstruction of other aspects of sound content (refs. ^55,128,129^)?
- What accounts for large inter-individual differences in several parameters of FFR sound encoding even in a homogeneous population of healthy, normal-hearing adults^76^? What are the neural origins and behavioral correlates of these differences?
- How does lateralization of cortical and subcortical FFR, observed in several studies^4,69,113^, emerge in the system, and what functional relevance does it have?
- What kinds of circuit dynamics do we expect as different structures interact, and can these be observed using FFR methods that allow for source separation? Can bottom-up and top down influences be definitively separated using the FFR?
- To what extent do FFR fundamental frequencies and harmonics reflect activity from different cortical and subcortical sources, and under which conditions of measurement (e.g. MEG vs. EEG, stimulus properties)? How and where is this information integrated in the brain?
- What are the neural origins of group differences and experience-related changes in the FFR? How does the relative contribution of brainstem, thalamic, and cortical sources vary as a function of age or experience with sound? Do some pathologies affect certain FFR generators more than others?
- Can we integrate predictions stemming from theoretical models of oscillatory activity (ref. ^116^) with physiological FFR data to achieve a better understanding of the functional properties of the whole system?
- Is the FFR the product of feed-forward transmission of phase-locked spiking, or alternatively, active oscillatory circuits?

## Conclusions

Auditory neuroscience is now more attuned to the significance of top-down influences and the role of neuroplasticity in auditory processing; the auditory system is correctly viewed as part of interconnected circuitry that involves cognitive, sensorimotor, and limbic systems. In many ways, the FFR is an ideal way to access this complex circuit precisely because it is not a monolithic response reflecting only a single stimulus component or single source. Rather, the FFR reveals how the auditory system responds to multiple acoustic elements throughout an entire sound, enabling a wealth of analysis strategies. Germane to this perspective article, the FFR can be measured with a number of different techniques, each of which provides a distinct window into auditory processing. Because the FFR is so rich and complex, much more is to be learned from it (Box 4). There needs to be agreement on terminology, a concerted effort against over-generalization vis-à-vis its generation, and careful harmonization between techniques and research questions to fully understand and successfully harness its potential. We hope this perspective piece serves to both inform readers and to inspire them to embrace the complexity of the FFR while remaining grounded in best practices and interpretation as research into the brain mechanisms underlying this response proceeds.

## References

[1] Schnupp, J., Nelken, I. & King, A. *Auditory Neuroscience Making Sense of Sound* (MIT Press, 2011).

[2] Skoe E, Kraus N (2010). Auditory brain stem response to complex sounds: a tutorial. Ear Hear..

[3] Krizman Jennifer, Kraus Nina (2019). Analyzing the FFR: A tutorial for decoding the richness of auditory function. Hearing Research.

[4] Coffey EBJJ, Herholz SC, Chepesiuk AMPP, Baillet S, Zatorre RJ (2016). Cortical contributions to the auditory frequency-following response revealed by MEG. Nat. Commun..

[5] Moushegian G, Rupert AL, Stillman RD (1973). Scalp-recorded early responses in man to frequencies in the speech range. Electroencephalogr. Clin. Neurophysiol..

[6] Kraus N, Nicol T (2017). The power of sound for brain health. Nat. Hum. Behav..

[7] Nozaradan S, Schönwiesner M, Caron-Desrochers L, Lehmann A (2016). Enhanced brainstem and cortical encoding of sound during synchronized movement. Neuroimage.

[8] Musacchia G, Sams M, Skoe E, Kraus N (2007). Musicians have enhanced subcortical auditory and audiovisual processing of speech and music. Proc. Natl. Acad. Sci. USA.

[9] Thompson EC, Woodruff Carr K, White-Schwoch T, Otto-Meyer S, Kraus N (2017). Individual differences in speech-in-noise perception parallel neural speech processing and attention in preschoolers. Hear. Res..

[10] Marmel F (2013). Subcortical neural synchrony and absolute thresholds predict frequency discrimination independently. J. Assoc. Res. Otolaryngol..

[11] Omote A, Jasmin K, Tierney A (2017). Successful non-native speech perception is linked to frequency following response phase consistency. Cortex.

[12] Zhao TC, Kuhl PK (2018). Linguistic effect on speech perception observed at the brainstem. Proc. Natl. Acad. Sci. USA.

[13] Krishnan A, Xu Y, Gandour J, Carianib P, Cariani P (2005). Encoding of pitch in the human brainstem is sensitive to language experience. Cogn. Brain Res..

[14] Wong PCM, Skoe E, Russo NM, Dees T, Kraus N (2007). Musical experience shapes human brainstem encoding of linguistic pitch patterns. Nat. Neurosci..

[15] Reetzke R, Xie Z, Llanos F, Chandrasekaran B (2018). Tracing the trajectory of sensory plasticity across different stages of speech learning in adulthood. Curr. Biol..

[16] Skoe E, Krizman J, Spitzer E, Kraus N (2013). The auditory brainstem is a barometer of rapid auditory learning. Neuroscience.

[17] Parbery-Clark A, Anderson S, Hittner E, Kraus N (2012). Musical experience offsets age-related delays in neural timing. Neurobiol. Aging.

[18] Krizman J, Marian V, Shook A, Skoe E, Kraus N (2012). Subcortical encoding of sound is enhanced in bilinguals and relates to executive function advantages. Proc. Natl. Acad. Sci. USA.

[19] Colella-Santos MF, Donadon C, Sanfins MD, Borges LR (2019). Otitis media: long-term effect on central auditory nervous system. Biomed. Res. Int..

[20] Elmer S, Hausheer M, Albrecht J, Kühnis J (2017). Human brainstem exhibits higher sensitivity and specificity than auditory-related cortex to short-term phonetic discrimination learning. Sci. Rep..

[21] Jafari Z, Malayeri S (2014). Effects of congenital blindness on the subcortical representation of speech cues. Neuroscience.

[22] Jeng FC (2011). Cross-linguistic comparison of frequency-following responses to voice pitch in american and chinese neonates and adults. Ear Hear..

[23] Presacco A, Simon JZ, Anderson S (2019). Speech-in-noise representation in the aging midbrain and cortex: effects of hearing loss. PLoS One.

[24] Daly DMD, Roeser RJR, Moushegian G, clinical GM-E (1976). and & 1976, undefined. The frequency-following response in subjects with profound unilateral hearing loss. Electronencephalogr. Clin. Neurophysiol..

[25] Zhong Z, Henry KS, Heinz MG (2014). Sensorineural hearing loss amplifies neural coding of envelope information in the central auditory system of chinchillas. Hear. Res..

[26] Shaheen LA, Valero MD, Liberman MC (2015). Towards a diagnosis of cochlear neuropathy with envelope following responses. J. Assoc. Res. Otolaryngol..

[27] Hornickel J, Kraus N (2013). Unstable representation of sound: a biological marker of dyslexia. J. Neurosci..

[28] White-Schwoch T (2015). Auditory processing in noise: a preschool biomarker for literacy. PLoS Biol..

[29] Chandrasekaran B, Hornickel J, Skoe E, Nicol T, Kraus N (2009). Context-dependent encoding in the human auditory brainstem relates to hearing speech in noise: implications for developmental dyslexia. Neuron.

[30] Basu M, Krishnan A, Weber-Fox C (2010). Brainstem correlates of temporal auditory processing in children with specific language impairment. Dev. Sci..

[31] Billiet CR, Bellis TJ (2010). The relationship between brainstem temporal processing and performance on tests of central auditory function in children with reading disorders. J. Speech Lang. Hear. Res..

[32] Rocha-Muniz CN, Befi-Lopes DM, Schochat E (2012). Investigation of auditory processing disorder and language impairment using the speech-evoked auditory brainstem response. Hear. Res..

[33] Otto-Meyer S, Krizman J, White-Schwoch T, Kraus N (2018). Children with autism spectrum disorder have unstable neural responses to sound. Exp. Brain Res..

[34] Russo N, Nicol T, Trommer B, Zecker S, Kraus N (2009). Brainstem transcription of speech is disrupted in children with autism spectrum disorders. Dev. Sci..

[35] Musacchia G (2018). Effects of noise and age on the infant brainstem response to speech. Clin. Neurophysiol..

[36] Ribas-Prats T (2019). The frequency-following response (FFR) to speech stimuli: a normative dataset in healthy newborns. Hear. Res..

[37] Lai J, Bartlett EL (2018). Masking differentially affects envelope-following responses in young and aged animals. Neuroscience.

[38] Parthasarathy A, Datta J, Torres JAL, Hopkins C, Bartlett EL (2014). Age-related changes in the relationship between auditory brainstem responses and envelope-following responses. J. Assoc. Res. Otolaryngol..

[39] Krizman J, Bonacina S, Kraus N (2019). Sex differences in subcortical auditory processing emerge across development. Hear. Res..

[40] Anderson S, White-Schwoch T, Parbery-Clark A, Kraus N (2013). Reversal of age-related neural timing delays with training. Proc. Natl. Acad. Sci. USA.

[41] Song J, Skoe E, Wong P, Kraus N (2008). Plasticity in the adult human auditory brainstem following short-term linguistic training. J. Cogn. Neurosci..

[42] Tierney AT, Krizman J, Kraus N (2015). Music training alters the course of adolescent auditory development. Proc. Natl. Acad. Sci. USA.

[43] Kraus N (2017). The neural legacy of a single concussion. Neurosci. Lett..

[44] Kraus N, White-Schwoch T (2015). Unraveling the biology of auditory learning: a cognitive-sensorimotor-reward framework. Trends Cogn. Sci..

[45] Kraus Nina, Anderson Samira, White-Schwoch Travis (2017). The Frequency-Following Response: A Window into Human Communication. The Frequency-Following Response.

[46] Bidelman GM (2018). Subcortical sources dominate the neuroelectric auditory frequency-following response to speech. Neuroimage.

[47] Zhang X, Gong Q (2019). Frequency-following responses to complex tones at different frequencies reflect different source configurations. Front. Neurosci..

[48] Worden F, Marsh J (1968). Frequency-following (microphonic-like) neural responses evoked by sound. Electroencephalogr. Clin. Neurophysiol..

[49] Galbraith, G. & Doan, B. Brainstem frequency-following and behavioral responses during selective attention to pure tone and missing fundamental stimuli. *Int. J. Psychophysiol.* **19**, 203–214 (1995). 10.1016/0167-8760(95)00008-g 7558987

[50] Galbraith GC, Jhaveri SP, Kuo J (1997). Speech-evoked brainstem frequency-following responses during verbal transformations due to word repetition. Electroencephalogr. Clin. Neurophysiol..

[51] Smith JC, Marsh JT, Brown WS (1975). Far-field recorded frequency-following responses: evidence for the locus of brainstem sources. Electroencephalogr. Clin. Neurophysiol..

[52] Sohmer H, Pratt H, Kinarti R (1977). Sources of frequency following responses (FFR) in man. Electroencephalogr. Clin. Neurophysiol..

[53] Stillman RD, Crow G, Moushegian G (1978). Components of the frequency-following potential in man. Electroencephalogr. Clin. Neurophysiol..

[54] Herdman AT (2002). Intracerebral sources of human auditory steady-state responses. Brain Topogr..

[55] Dean Linden R, Picton TW, Hamel G, Campbell KB (1987). Human auditory steady-state evoked potentials during selective attention. Electroencephalogr. Clin. Neurophysiol..

[56] Pérez-González D, Malmierca MS, Covey E (2005). Novelty detector neurons in the mammalian auditory midbrain. Eur. J. Neurosci..

[57] Shiga T (2015). Deviance-related responses along the auditory hierarchy: combined FFR, MLR and MMN evidence. PLoS One.

[58] Skoe E, Krizman J, Spitzer E, Kraus N (2015). Prior experience biases subcortical sensitivity to sound patterns. J. Cogn. Neurosci..

[59] Carbajal GV, Malmierca MS (2018). The neuronal basis of predictive coding along the auditory pathway: from the subcortical roots to cortical deviance detection. Trends Hear..

[60] Chandrasekaran B, Kraus N (2010). The scalp-recorded brainstem response to speech: neural origins and plasticity. Psychophysiology.

[61] Kiren T, Aoyagi M, Furuse H, Koike Y (1994). An experimental study on the generator of amplitude-modulation following response. Acta Otolaryngol. Suppl..

[62] Kuwada S (2002). Sources of the scalp-recorded amplitude-modulation following response. J. Am. Acad. Audiol..

[63] White-Schwoch T, Nicol T, Warrier CM, Abrams DA, Kraus N (2017). Individual differences in human auditory processing: insights from single-trial auditory midbrain activity in an animal model. Cereb. Cortex.

[64] King A, Hopkins K, Plack CJ (2016). Differential group delay of the frequency following response measured vertically and horizontally. J. Assoc. Res. Otolaryngol..

[65] Akhoun I (2008). The temporal relationship between speech auditory brainstem responses and the acoustic pattern of the phoneme/ba/in normal-hearing adults. Clin. Neurophysiol..

[66] Brugge JF (2008). Functional localization of auditory cortical fields of human: click-train stimulation. Hear. Res..

[67] Galbraith GC (1994). Two-channel brain-stem frequency-following responses to pure tone and missing fundamental stimuli. Electroencephalogr Clin. Neurophysiol. Potentials Sect..

[68] Zhang Xiaochen, Gong Qin (2017). Correlation between the frequency difference limen and an index based on principal component analysis of the frequency-following response of normal hearing listeners. Hearing Research.

[69] Coffey EBJ, Musacchia G, Zatorre RJ (2016). Cortical correlates of the auditory frequency-following and onset responses: EEG and fMRI evidence. J. Neurosci..

[70] Coffey EBJ, Chepesiuk AMP, Herholz SC, Baillet S, Zatorre RJ (2017). Neural correlates of early sound encoding and their relationship to speech-in-noise perception. Front. Neurosci..

[71] Hartmann Thomas, Weisz Nathan (2019). Auditory cortical generators of the Frequency Following Response are modulated by intermodal attention. NeuroImage.

[72] Tichko P, Skoe E (2017). Frequency-dependent fine structure in the frequency-following response: the byproduct of multiple generators. Hear. Res..

[73] Lin F-H (2006). Assessing and improving the spatial accuracy in MEG source localization by depth-weighted minimum-norm estimates. Neuroimage.

[74] Baillet S (2017). Magnetoencephalography for brain electrophysiology and imaging. Nat. Neurosci..

[75] Gross J (2013). Good practice for conducting and reporting MEG research. Neuroimage.

[76] Coffey EBJ, Colagrosso EMG, Lehmann A, Schönwiesner M, Zatorre RJ (2016). Individual differences in the frequency-following response: relation to pitch perception. PLoS One.

[77] Galbraith GC (2000). Putative measure of peripheral and brainstem frequency-following in humans. Neurosci. Lett..

[78] Bidelman GM, Davis MK, Pridgen MH (2018). Brainstem-cortical functional connectivity for speech is differentially challenged by noise and reverberation. Hear. Res..

[79] Musacchia G, Strait DL, Kraus N (2008). Relationships between behavior, brainstem and cortical encoding of seen and heard speech in musicians and non-musicians. Hear. Res..

[80] Presacco Alessandro, Simon Jonathan Z., Anderson Samira (2016). Effect of informational content of noise on speech representation in the aging midbrain and cortex. Journal of Neurophysiology.

[81] Bastos AM, Schoffelen J-M (2016). A tutorial review of functional connectivity analysis methods and their interpretational pitfalls. Front. Syst. Neurosci..

[82] Chandrasekaran B, Kraus N, Wong PCM (2012). Human inferior colliculus activity relates to individual differences in spoken language learning. J. Neurophysiol..

[83] Ayala YA, Lehmann A, Merchant H (2017). Monkeys share the neurophysiological basis for encoding sound periodicities captured by the frequency-following response with humans. Sci. Rep..

[84] Warrier CM, Abrams DA, Nicol TG, Kraus N (2011). Inferior colliculus contributions to phase encoding of stop consonants in an animal model. Hear. Res..

[85] Abrams DA, Nicol T, White-Schwoch T, Zecker S, Kraus N (2017). Population responses in primary auditory cortex simultaneously represent the temporal envelope and periodicity features in natural speech. Hear. Res..

[86] Behroozmand R (2016). Neural correlates of vocal production and motor control in human Heschl’s gyrus. J. Neurosci..

[87] Puschmann Sebastian, Baillet Sylvain, Zatorre Robert J (2018). Musicians at the Cocktail Party: Neural Substrates of Musical Training During Selective Listening in Multispeaker Situations. Cerebral Cortex.

[88] Forte, A. E., Etard, O. & Reichenbach, T. The human auditory brainstem response to running speech reveals a subcortical mechanism for selective attention. *Elife* **6**, 1–12 (2017). 10.7554/eLife.27203 PMC5634786 28992445

[89] Maddox, R. K. & Lee, A. K. C. Auditory brainstem responses to continuous natural speech in human listeners. eNeuro **5**, ENEURO.0441-17.2018 **5**, 1–13 (2018). 10.1523/ENEURO.0441-17.2018 PMC5806592 29435487

[90] Etard O, Kegler M, Braiman C, Forte AE, Reichenbach T (2019). Decoding of selective attention to continuous speech from the human auditory brainstem response. Neuroimage.

[91] Gama N, Peretz I, Lehmann A (2016). Recording the human brainstem frequency-following-response in the free-field. J. Neurosci. Methods.

[92] Kraus N, Hornickel J, Strait DL, Slater J, Thompson E (2014). Engagement in community music classes sparks neuroplasticity and language development in children from disadvantaged backgrounds. Front. Psychol..

[93] Wiegers JS, Bielefeld EC, Whitelaw GM (2015). Utility of the Vivosonic IntegrityTM auditory brainstem response system as a hearing screening device for difficult-to-test children. Int. J. Audiol..

[94] Yi HG, Xie Z, Reetzke R, Dimakis AG, Chandrasekaran B (2017). Vowel decoding from single-trial speech-evoked electrophysiological responses: a feature-based machine learning approach. Brain Behav..

[95] Xie Z, Reetzke R, Chandrasekaran B (2019). Machine learning approaches to analyze speech-evoked neurophysiological responses. J. Speech Lang. Hear. Res..

[96] Mišić B, Sporns O (2016). From regions to connections and networks: New bridges between brain and behavior. Curr. Opin. Neurobiol..

[97] Griffiths TD, Warren JD (2002). The planum temporale as a computational hub. Trends Neurosci..

[98] Hickok G, Poeppel D (2007). The cortical organization of speech processing. Nat. Rev. Neurosci..

[99] Rauschecker J, Scott S (2009). Maps and streams in the auditory cortex: nonhuman primates illuminate human speech processing. Nat. Neurosci..

[100] Zatorre RJ, Chen J, Penhune V (2007). When the brain plays music: auditory–motor interactions in music perception and production. Nat. Rev. Neurosci..

[101] Feng Gangyi, Yi Han Gyol, Chandrasekaran Bharath (2018). The Role of the Human Auditory Corticostriatal Network in Speech Learning. Cerebral Cortex.

[102] Bidelman GM, Villafuerte JW, Moreno S, Alain C (2014). Age-related changes in the subcortical–cortical encoding and categorical perception of speech. Neurobiol. Aging.

[103] Holmes E, Purcell DW, Carlyon RP, Gockel HE, Johnsrude IS (2018). Attentional modulation of envelope-following responses at lower (93–109 Hz) but not higher (217–233 Hz) modulation rates. J. Assoc. Res. Otolaryngol..

[104] Hoormann J, Falkenstein M, Hohnsbein J (2004). Effects of spatial attention on the brain stem frequency-following potential. Neuroreport.

[105] Lehmann A, Schönwiesner M (2014). Selective attention modulates human auditory brainstem responses: relative contributions of frequency and spatial cues. PLoS One.

[106] Varghese L, Bharadwaj HM, Shinn-Cunningham BG (2015). Evidence against attentional state modulating scalp-recorded auditory brainstem steady-state responses. Brain Res..

[107] Mai G, Schoof T, Howell P (2019). Modulation of phase-locked neural responses to speech during different arousal states is age-dependent. Neuroimage.

[108] Hairston WD, Letowski TR, McDowell K (2013). Task-related suppression of the brainstem frequency following response. PLoS One.

[109] Ulanovsky N, Las L, Nelken I (2003). Processing of low-probability sounds by cortical neurons. Nat. Neurosci..

[110] King C, McGee T, Rubel EW, Nicol T, Kraus N (1995). Acoustic features and acoustic change are represented by different central pathways. Hear. Res..

[111] Parras GG (2017). Neurons along the auditory pathway exhibit a hierarchical organization of prediction error. Nat. Commun..

[112] Nozaradan S (2014). Exploring how musical rhythm entrains brain activity with electroencephalogram frequency-tagging. Philos. Trans. R. Soc. B.

[113] Hornickel J, Skoe E, Kraus N (2009). Subcortical laterality of speech encoding. Audiol. Neurootol..

[114] Bharadwaj HM, Shinn-Cunningham BG (2014). Rapid acquisition of auditory subcortical steady state responses using multichannel recordings. Clin. Neurophysiol..

[115] Aiken SJ, Picton TW (2008). Envelope and spectral frequency-following responses to vowel sounds. Hear. Res..

[116] Lerud KD, Almonte FV, Kim JC, Large EW (2014). Mode-locking neurodynamics predict human auditory brainstem responses to musical intervals. Hear. Res..

[117] Luo L, Wang Q, Li L (2017). Neural representations of concurrent sounds with overlapping spectra in rat inferior colliculus: comparisons between temporal-fine structure and envelope. Hear. Res..

[118] Joris PX, Schreiner CE, Rees A (2004). Neural processing of amplitude-modulated sounds. Physiol. Rev..

[119] Moller HJ, Devins GM, Shen J, Shapiro CM (2006). Sleepiness is not the inverse of alertness: evidence from four sleep disorder patient groups. Exp. Brain Res..

[120] Wang X, Lu T, Bendor D, Bartlett E (2008). Neural coding of temporal information in auditory thalamus and cortex. Neuroscience.

[121] Rouiller E, de Ribaupierre Y, de Ribaupierre F (1979). Phase-locked responses to low frequency tones in the medial geniculate body. Hear. Res..

[122] Brugge JF (2009). Coding of repetitive transients by auditory cortex on Heschl’s gyrus. J. Neurophysiol..

[123] Nourski KV (2013). Coding of repetitive transients by auditory cortex on posterolateral superior temporal gyrus in humans: an intracranial electrophysiology study. J. Neurophysiol..

[124] Irvine, D. R. F. The auditory brainstem: a review of the structure and function of auditory brainstem processing mechanisms. In *Progress in Sensory Physiology*, Vol. 7 (ed Ottoson, D.) (Springer-Verlag, Berlin, 1986).

[125] Steinschneider M, Arezzo J, Vaughan HG (1980). Phase-locked cortical responses to a human speech sound and low-frequency tones in the monkey. Brain Res..

[126] Wallace MN, Shackleton TM, Palmer AR (2002). Phase-locked responses to pure tones in the primary auditory cortex. Hear. Res..

[127] Batra R, Kuwada S, Maher VL (1986). The frequency-following response to continuous tones in humans. Hear. Res..

[128] Anumanchipalli GK, Chartier J, Chang EF (2019). Speech synthesis from neural decoding of spoken sentences. Nature.

[129] Ding N, Simon JZ (2012). Emergence of neural encoding of auditory objects while listening to competing speakers. Proc. Natl Acad. Sci. USA.

